# Ethical aspects of vulnerability in research

**DOI:** 10.1007/s10202-012-0109-2

**Published:** 2012-10-17

**Authors:** Elisabeth Weisser-Lohmann

**Affiliations:** Hagen, Germany

## Abstract

In connection with research on humans, the term “vulnerability” is only appropriate to identify the special need for protection of certain sections of the population and individuals, if this term refers to the additional risk of certain groups of subjects. Authors who focus on the additional risk suffering of a subject group when defining vulnerability succeed in considering the specific worthiness of protection in a context-sensitive way. The attempt to define the risk–benefit assessment for vulnerable subject groups on a binding basis faces considerable difficulties. This assessment depends both on the research situation and on the test subject. The normative aspect of this decision could be solved by referring to Rawl’s decision model of an original position. In cases where there is no benefit for the subject, arguments in the discussion of the risks and benefits that are based on a “group or overall benefit” and an “objective interest,” cannot be fully sustained.

The principle of equality requires treating equal cases with the same rules. However, in societal supply systems like the healthcare system the principle of equality does not aim at equality in the descriptive sense: As a normative postulate “equality” substitutes, descriptively speaking, unequal claimants for basically equal claimants. A violation of this principle of equality is given if differences between the members of a society inhibit access to economic resources, health care, education and political participation. This exclusion finds very little acceptance if the impeded access is based on differences that cannot be attributed to the actors themselves: where discrimination is due to physical or psychic restrictions, origin, age or social position. The term often used here is “vulnerable groups,” and their special need for protection is generally accepted.

Apart from the right to life (Art. 6) and the right to personal freedom (Art. 9), in Article 7 in the international covenant of 16th December 1966 on civil and political rights (Uno-Covenant II) we also find established: “No one shall be subjected without his free consent to medical or scientific experimentation” (Office of the United Nations High Commissioner for Human Rights 2012).

In the past, however, this protection often let to situations in which sections of the population incapable of making or approving decisions were excluded from participating in the diagnostic and therapeutic progress of medical services. As an example, we can refer to the highly critical situation of pharmaceutical supply for children: For the majority of the available pharmaceutics, there are no or insufficient data for the use in pediatrics. The off-label prescription of pharmaceutics is up to 80 % depending on age category and place (more frequent with younger patients, a higher rate in hospitals) (Lenk et al. 2011).

The example shows that the recognition of the special need for protection of vulnerable sections of the population does by no means explain how to meet the requirements, without taking into account severe disadvantages which again exclude this group. A way out of the dilemma (Beauchamp and Childress 2001:10)^1^ can only be achieved by defining vulnerability in certain contexts. For it is by no means sufficient to simply state that “[p]regnant women are a vulnerable section of the population” to meet the special claim of protection in certain contexts. In fact, claims and obligations resulting from the “vulnerability” of a group can only be obtained by clarifying the normative content of this term.

This clarification first of all requires differentiating between the general use of “vulnerable” in the sense of “needy” and the use of the term in the context of research on humans. In everyday language, for instance, we talk about the neediness of a blind person as a consequence of his or her restricted capacity to defend him- or herself. This neediness in everyday life is different from the one in a research context, for obviously the neediness of a blind human does not define him as a vulnerable subject of research. Thus, the question must be: In what way are certain individuals or groups defined as vulnerable groups in a research context?

Almost without exception, recent regulations in biomedical research on humans are based on the rule of informed consent, derived from the principle of autonomy. In this context, “vulnerability” is understood as the “incapacity or limited capacity of consent of a group of patients.” The Helsinki declaration revised in 2008 confirms the principle of free consent after sufficient explanation as a compulsory requirement that research projects on humans have to fulfill. This principle is extended by regulations which are to allow controlled research also on “incompetent persons,” “persons susceptible to coercion,” “persons who will not derive direct benefits from participation” and “persons for whom research is mixed with clinical care.” The Council for International Organizations of Medical Sciences (CIOMS), supported by the World Health Organization and the UNESCO, adds to the group of vulnerable persons “those with limited capacity or freedom to consent or to decline to consent […] children, and persons who because of mental or behavioral disorders are incapable of giving informed consent.” This definition is specified by the additions:

junior or subordinate members of a hierarchical group […] as medical and nursing students, subordinate hospital and laboratory personnel, employers of pharmaceutical companies, and members of the armed forces or police, elderly persons, the unemployed, homeless persons, nomads, prisoners, members of communities unfamiliar with modern medical concepts […].

This enumeration points out as follows: The characteristic “incapacity or limited capacity of consent” is in fact a necessary but not at all sufficient condition for defining the vulnerability of a section of the population. What does become clear is that there are situations in which we cannot expect “normal” patients or subjects to represent their interests in an acceptable way. That is why further criteria have to be considered when ethically reviewing the reliability of research on humans. The relevant regulations, however, refrain from designating these criteria, but they replace them with the enumeration of vulnerable sections of the population. This approach not only suggests that the list could be expanded as desired but it moreover leads to undermining the normative demand so that the required special claim for protection remains unclear. This situation can only be remedied by a closer definition of the concept of vulnerability of certain sections of the population in view of biomedical research on humans. Only on that basis, ethical criteria for research projects can be identified.

The proposals can especially be distinguished by their differing range: “Vulnerability” can serve to identify particular sections of the population but also to characterize life as a whole and—as a basic anthropological classification—it can demand each life’s worthiness of protection (Callahan 2000). In the context of research, both the narrower and the broader definitions are not expedient (Kottow 2005). Some authors concentrate on damage or harm in defining vulnerability. On the basis of human neediness, the expectable additional harm becomes a criterion for the identification of vulnerable groups. This suggestion uncovers an important ambiguity of the term “vulnerable”: If it is said that men are a vulnerable group for suffering from testicle cancer, the underlying idea is that only men can be affected by this disease. The affiliation to the group of male individuals is a presumption for this disease. The increased risk to suffer from a disease has to be differentiated from this use of the word: Thus, men belonging to a certain occupational group bear a larger risk to suffer from this kind of cancer. Only this additional higher risk defines this section of the population as vulnerable in the context considered here. However, individuals who—due to their social status—bear a higher risk of an invalid consent are not included in this definition (e.g., dependent occupational groups, inmates of institutions).

A combination of the definitions “capable of consent” and “harm” is to rectify the deficiency. Vulnerable are those individuals “whose capacity to safeguard their own interests as research participants, through the process of informed consent or refusal, is compromised” (Nickel 2006:248). If in this case, the principle of consent is combined with the principle of a fair consideration of individual interests, it indeed remains unclear who will decide about the respective interests in each case. The definition violates the principles of equality and freedom as it remains unclear who determines what can be regarded as one’s own interest and what importance can be attached to the objective interests in this context.

Agrawal (2003:25) avoids these difficulties by focusing not on the interests but on the obligation to protect which is linked to the “principle of vulnerability”: Vulnerability is an “increased potential that one’s interest cannot be protected.” The greater obligation to protect, which is based on vulnerability, is connected with the occurrence probability of additional or larger calamities. Hence, S. Hurst understands vulnerability to be the obligation to special protection against “identifiably increased likelihood of incurring additional or greater wrong” (Hurst 2008:195). The vulnerability of a group describes the right to special protection and thus is based on the identifiably increased likelihood of the occurrence of damage for the members of this group and generates the right to be protected against this higher risk. This right is subject to the obligation to prevent any damage. In view of the causes for the increased likelihood to suffer a calamity, we differentiate between “extrinsic” and “intrinsic” vulnerability. If the higher risk is due to external circumstances such as financial limitations, lack of education, etc., we call it extrinsic vulnerability; if the higher risk is due to individual characteristics such as illness, physical or mental handicaps or age, we call it intrinsic vulnerability. In this definition, it is especially the parameter “identifiably increased likelihood” which allows rendering the term “vulnerability” more precisely in the context of examining the ethical acceptability of research with or on humans.

The following risks have to be taken into consideration with biomedical research projects on humans and with obtaining the informed consent or with examining the project: (1) the risk of physical harm, (2) the risk of mental harm, (3) the risk of social impairment and stigmatization as well as (4) the risk of financial burdens due to costs connected with the participation.

With subjects having no, a limited or a “wrong” consent, an external examination of the evaluation of risks and burdens has to be carried out. It takes place according to the principle of proportionality between the possible risks and the burdens for the subject and the benefit to be expected as well as by considering the increased likeliness of additional suffering. When defining the risks, three aspects have to be differentiated: (1) How likely is it that there will be additional harm as compared with the “normal” group of subjects? (2) How severe is the harm? (3) Is it reasonable at all to harm the subject? Parameters 1 and 2 have to be specified on the basis of the criterion “vulnerability” developed here. However, to evaluate whether such risks are reasonable requires a reliable standard. It is definitely problematic to draw a comparison with the dangers of everyday life (Spriggs 2004:179) because on the one hand—with regard to likeliness of occurrence and severity—there is often a considerable difference between the various dangers of everyday life and on the other hand it is by no means clear what can be defined as “dangers of everyday life.” And even the normative question if it is acceptable to expose vulnerable subjects to additional risks of everyday life has not been settled at all. This accusation also applies to the attempt to quantify minimal risks and burdens modeled after risks in everyday life (e.g., mortality risk in road traffic, risk of sport injuries) (Wendler et al. 2005). In view of the special situation of research with children, Nelson and Ross suggest measuring the reasonableness of impairments by asking which mental and physical burdens conscientious parents may place on their children for pedagogical reasons (Ross and Nelson 2005:759). But even this procedure does not lead to a generally binding definition of reasonable risks. A decision on that point depends both on the research situation and on the subjects. But, as suggested by Ross and Nelson, the normative aspect of this decision could certainly be solved by Rawl’s decision model of an original position: The decision on the reasonableness of an increased risk simulates a situation of not knowing, in which the decision maker does not discern, if he or she or a person for whom he or she is responsible should participate in a research project. What kind of increased risk leads to the decision that someone or a person entrusted to someone’s care would abstain from taking part? This decision would always take into account the expected benefits since the question of the potential risk depends on the degree and type of benefit: According to the principle of proportionality between risk and benefit, a greater risk can be accepted if with a participation life could be saved or a considerable improvement of the living quality could be achieved.

In any case, it is a problematic question if carrying out research projects with sections of the population with a comparatively higher risk of harm as compared to other subjects is acceptable even if there is no individual benefit. If the group of subjects do not benefit from the research project, then it is not for their own good so that, as Seelmann (2002) objects, these subjects are instrumentalized in favor of a third party which does not justify an injury or the higher risk of an injury. G. Maio suggests to speak of an only partial instrumentalization.^2^ Partial instrumentalizations can be compared with everyday situations in which people are involved in certain functional connections which are by no means regarded as a violation of human dignity. If, in this connection, one often mentions the benefit to be expected for the group, there is still a conflict between two competing obligations. The negative obligation to avoid instrumentalization is opposed to the positive obligation to help sick children in the future. From a legal point of view, the obligation to avoid instrumentalization is more important as it is directly based on the recognition of the fundamental rights of others (Maio 2010:52).^3^ Research on vulnerable groups of subjects beyond a therapeutic context referring to a “benefit for the group” or an objective interest seems to be problematic against this background. The argument of a group benefit after all presupposes that the subject can be expected to solidarize with this group or its interests by his or her participation. This may succeed, above all, if the group or overall benefit can be understood in the sense of one’s own expected benefit and if the whole is constituted by identifiers such as common age or diseases. However, as a decisive argument for the admission of certain subject groups, this understanding of an overall benefit is problematic: The suspicion of a hidden use for others can hardly be removed in this way. To assume an “objective interest” like the progress in medicine implies that this is an interest that can be attributed to everybody. Yet, this assumption remains hypothetical and can always be falsified by a defensive attitude of the subject in any given case. The term “vulnerability” as an instrument for specifying a particular claim of protection is stretched to its limits in a research profile, in which the “normal risk” like the additional risk is not compensated by any benefit.

To sum it up “Vulnerability” is not an individual normative term which generates certain rights and obligations. Instead, this term is based on the right to physical integrity. “Vulnerability” constitutes a special worthiness of protection measured in terms of the additional risk of suffering of a particular group of subjects. In cases when this obligation to protect leaves room for a risk–benefit assessment, this additional risk has to be taken into account. The obligation’s addressees are all persons involved in a research project. Perhaps Rawl’s decision model of an original position can provide guidance by using the veil of ignorance: This means that those who have to take a decision fictitiously assume the role of those individuals who do not know which role they will have to play in the context of the research project. With this procedure that blinds out the currently represented interests without losing sight of the existing interests, all the persons’ interests involved are taken into consideration in an appropriate way, but especially those of the future subjects.

## Footnotes

1 Beauchamp and Childress use dilemmas in the sense of contradictory findings motivated by actions (Beauchamp and Childress 2001:10).

2 Compare the statement of K. Seelmann in: Schwarz oder Weiss. In: Bioethica Forum Volume 53, 9–10.

3 Compare also id., Ethik der Forschung am Menschen. Stuttgart-Bad Cannstatt 2002, as well as id., Schwarz oder Weiss. In: Bioethica Forum. Volume 53 (2007), 6–10.
